# DNA barcoding of *Messor* ants of Bulgaria with insights into their taxonomic diversity

**DOI:** 10.3897/BDJ.13.e168586

**Published:** 2025-11-04

**Authors:** Albena Lapeva-Gjonova, Monika Pramatarova, Lech Borowiec, Ilia Gjonov, Rumyana Kostova, Rostislav Bekchiev, Simeon Borissov

**Affiliations:** 1 Sofia University, Faculty of Biology, Sofia, Bulgaria Sofia University, Faculty of Biology Sofia Bulgaria; 2 National Museum of Natural History, Bulgarian Academy of Sciences, Sofia, Bulgaria National Museum of Natural History, Bulgarian Academy of Sciences Sofia Bulgaria; 3 University of Wroclaw, Wroclaw, Poland University of Wroclaw Wroclaw Poland; 4 Institute of Biodiversity and Ecosystem Research, Bulgarian Academy of Sciences, Sofia, Bulgaria Institute of Biodiversity and Ecosystem Research, Bulgarian Academy of Sciences Sofia Bulgaria

**Keywords:** the Balkans, COI, species delimitation, Formicidae, Myrmicinae

## Abstract

**Background:**

Despite ongoing efforts to catalogue European ant species, studies focusing on the genetic diversity of Balkan ants remain limited. An integrative approach combining morphology, genetics, ecology and biogeography is preferable for accurately identifying species and resolving taxonomic uncertainties, particularly amongst challenging insect taxa, such as the ants in the genus *Messor* (Hymenoptera, Formicidae).

**New information:**

In this study, we analyse ants of the genus *Messor* using DNA barcode sequences, with a particular focus on the Bulgarian fauna. A total of 85 COI sequences were examined, including 84 from *Messor* specimens and one from *Aphaenogaster*, which was used as an outgroup. Of these, 81 sequences were newly generated, while four were retrieved from GenBank. The majority of specimens were collected in Bulgaria (61), with additional samples from Greece (13), Türkiye (4), Albania (1) and North Macedonia (2), providing broader genetic and geographic representation.

Althogether, 11 *Messor* morphospecies were identified, based on specimens used for molecular analysis. To assess the degree of congruence between morphological and molecular data, six species delimitation analyses were conducted: RESL, GMYC, ASAP, ABGD, bPTP and mPTP. In addition, haplotype network analysis of all sequences identified 35 distinct and coherently clustered haplotypes, providing insights into genetic diversity.

The COI barcode region successfully distinguished *Messor wasmanni* Krausse, 1910, *M. oertzeni* Forel, 1910 and *M. ibericus* Santschi, 1931. In contrast, species pairs, such as *M. atanassovii* Atanassov, 1982 and *M. creticus* Salata & Borowiec, 2019, as well as *M. ponticus* Steiner et al., 2018 and *M. hellenius* Agosti & Collingwood, 1987, could not be reliably differentiated using COI data. Furthermore, *Messor structor* (Latreille, 1798) showed high intraspecific genetic diversity. Finally, the *structor* and *instabilis* species groups were recovered with moderate to high support in both Maximum Likelihood and Bayesian Inference analyses, confirming that *M. oertzeni* and *M. hellenius* belong to the *structor* group.

Our results provide a reference for future research and underscore the value of integrative taxonomic approaches in ant biodiversity studies.

## Introduction

The genus *Messor*, commonly known as harvester ants, consists of typically granivorous species involved in seed dispersal, nutrient cycling and microclimate modification in surface soil layers (Cammeraat et al. 2002, Plowes et al. 2013, El Boukhrissi et al. 2023). These ants typically inhabit arid and semi-arid environments, with 134 species currently recognised within the Palaearctic, Afrotropical and Oriental biogeographic regions (Branstetter et al. 2022, Salata et al. 2023, Bolton 2025). While species richness is highest in North Africa and the Middle East, the environmental conditions and biogeographic history of the Southern Balkans also favour the presence of a substantial number of *Messor* species (Borowiec 2014, Janicki et al. 2016, Guénard et al. 2017, Wang et al. 2023, Juvé et al. 2025a).

Comprehensive modern studies on the species composition and distribution of *Messor* species in Bulgaria remain insufficient. Two recent species revisions have addressed some species within the genus found in the country. One such revision, focusing on the European species of the *structor* group (Steiner et al. 2018), included Bulgarian material, although samples originated from only a few localities. This study recognised three species from the group in Bulgaria: *Messor structor* (Latreille, 1798), *M. ponticus* Steiner et al., 2018 (with type locality in Bulgaria) and *M. ibericus* Santschi, 1931. Additionally, two other species, *M. mcarthuri* Steiner et al., 2018 and *M. hellenius* Agosti & Collingwood, 1987, were recently reported from the country by Lapeva-Gjonova and Borowiec (2022). The latter was not included in the revision of this group by Steiner et al. (2018), but was recognised as such by Borowiec and Salata (2025). Furthermore, a phylogenetic analysis by Juvé et al. (2025a) revealed *Messor oertzeni* Forel, 1910, a well-known species from Bulgaria, as the sixth member of the *structor* group in the country. *Messor wasmanni* Krausse, 1910 and *M. atanassovii* Atanassov, 1982, the latter with its type locality in Bulgaria, are the only representatives of the *instabilis* group currently known from Bulgaria. This species group from the Eastern Mediterranean region was recently revised (Salata et al. 2023). The revision included a detailed re-description of *M. atanassovii*, confirming its validity as a distinct species and reporting additional localities in both Bulgaria and Greece. The latest studies on *Messor* in the Palaearctic Region have re-evaluated the earlier records and concluded that four species previously reported from Bulgaria (*M. barbarus* (Linnaeus, 1767), *M. caducus* (Victor, 1839), *M. capitatus* (Latreille, 1798) and *M. concolor* Santschi, 1927) do not actually occur in the Balkans. Consequently, eight *Messor* species are currently recognised in Bulgaria: *M. atanassovii*, *M. wasmanni*, *M. oertzeni*, *M. structor*, *M. mcarthuri*, *M. ponticus*, *M. hellenius* and *M. ibericus* (Lapeva-Gjonova and Antonova 2022).

The scarcity of historical descriptions, coupled with high morphological variability within species and occurrences of hybridisation and even xenoparity, makes the genus *Messor* taxonomically and biologically challenging (Schlick-Steiner et al. 2006, Steiner et al. 2011, Romiguier et al. 2017, Steiner et al. 2018, Saar et al. 2023, Juvé et al. 2025a, Juvé et al. 2025b). This necessitates the application of complex approaches alongside the morphological one to resolve species delimitations.

DNA barcoding using mitochondrial cytochrome c oxidase I (COI) gene fragments has proven to be an efficient method for species identification and biodiversity assessment, including ants of *Messor* genus (Schlick-Steiner et al. 2006, Steiner et al. 2018, Strohmaier et al. 2025) and other Stenammini (Centorame et al. 2018, Gómez et al. 2018, Galkowski et al. 2019, Schifani et al. 2022, Zięcina et al. 2024). However, COI is not universally reliable for ant identification and species delimitation due to biological factors, such as incomplete lineage sorting, introgression, hybridisation, NUMTs and endosymbiont effects, as well as technical issues like gaps in reference libraries and threshold inconsistencies (Hurst and Jiggins 2005, Darras and Aron 2015, Romiguier et al. 2017). Despite these limitations, it remains a rapid, cost-effective tool with reasonable species-level resolution, supported by widely-used primers and extensive sequence repositories (deWaard et al. 2019, Martoni et al. 2024, Onyinyechi et al. 2025).

Accordingly, expanding barcoding efforts in underexplored regions, such as the Balkans, is crucial for improving our understanding of species diversity and evolutionary relationships. While a large-scale barcoding project of European ants is underway (Menchetti et al., unpublished), further research specifically targeting the genetic diversity of Balkan *Messor* ants will provide valuable insights into taxonomy and phylogeny.

## Sampling methods

### Sampling description

Specimens for DNA barcoding were primarily selected, based on morphology and their origin from diverse collection sites across the country. Morphological identifications followed Steiner et al. (2018) and Borowiec and Salata (2025).

**Molecular analyses**: DNA extraction, amplification and sequencing of the standard 658 bp COI barcode region were performed by the Canadian Centre for DNA Barcoding (CCDB) using the primers LepF1 and LepR1 (Hebert et al. 2004). DNA was extracted from the hind legs of specimens preserved in ethanol. In total, 84 COI *Messor* sequences were analysed, of which 80 were newly generated. The following four sequences were obtained from GenBank and were included in the phylogenetic analyses: KT184551 (*Messor structor*), KT184569 (*M. mcarthuri*), KT184511 (*M. ibericus*) from Steiner et al. (2018) and DQ074353 (*M. ponticus*) from Schlick-Steiner et al. (2006). The sequence of *Aphaenogaster festae* Emery, 1915 generated in the current study was selected as an outgroup in the phylogenetic analyses. All 81 sequences generated in this study are deposited in the Barcode of Life Data System (BOLD) under the BGMES project, where collection information and photos of each specimen are also provided. Voucher specimens are preserved in the Zoological Collection of Sofia University (BFUS).

To assess the degree of congruence between morphological identification conducted prior to the molecular data, multiple species delimitation approaches were applied to the molecular dataset. Sequence alignment and trimming were performed using MEGA v.12 (Kumar et al. 2024). In the BOLD system, the sequences were assigned to Barcode Index Numbers (BINs), an algorithm-based approach to delineate operational taxonomic units, which were automatically calculated for records by Refined Single Linkage (RESL) analysis. These BINs have a unique identifier and provide a good proxy for species (Ratnasingham and Hebert 2013). To estimate genetic distances and enable comparison, pairwise distances were calculated under the Kimura 2-parameter (K2P) model using MEGA v.12 (Kumar et al. 2024), whereafter species boundaries were tested with Assemble Species by Automatic Partitioning (ASAP) and Automatic Barcode Gap Discovery (ABGD). Subsequently, ultrametric trees were generated in BEAST v. 10.5.0 (Baele et al. 2025) with a strict clock, coalescent tree prior and 100 million generations, sampling every 1000 trees. The effective sample size (ESS) was monitored in Tracer v. 1.7.2 (Rambaut et al. 2018). Trees were summarised via TreeAnnotator (Suchard et al. 2018) removing 10% as a burn-in. Species delimitation analyses included Generalised Mixed Yule Coalescent Approach (GMYC) with a single threshold (implemented on the web server https://species.h-its.org/gmyc/, accessed on 27 July 2025), the Poisson Tree Processes (bPTP) (implemented on the web server http://species.h-its.org/ptp/, accessed on 28 July 2025) and, finally, the multi-rate Poisson Tree Processes (mPTP) (implemented on the web server http://mptp.h-its.org/#/tree, accessed on 27 July 2025) (Fujisawa and Barraclough 2013, Zhang et al. 2013, Trifinopoulos et al. 2016, Kapli et al. 2017).

Phylogenetic reconstruction was performed using both Maximum Likelihood (ML) and the Bayesian Inference (BI) analyses. ML analysis was performed in IQ-TREE (Nguyen et al. 2015) on the W-IQ-TREE interface (Trifinopoulos et al. 2016). The integrated ModelFinder (Kalyaanamoorthy et al. 2017) was used to infer the best substitution model. Nodal support was obtained through a standard non-parametric bootstrap with 1000 replicates. BI analysis was run using MrBayes v.3.2.7a (Ronquist et al. 2012). Phylogenetic trees (BI and ML) were visualised using iTOL v.5 (Letunic and Bork 2021). Haplotype analysis was conducted utilising the DnaSP v.6 software (Rozas et al. 2017) and the results were visualised through the utilisation of PopArt employing TCS network analysis (Clement et al. 2002, Leigh and Bryant 2015).

A map of sequence sampling sites was created in QGIS version 3.34.12-Prizren, based on the Cross Blended Hypsometric map layer (https://www.naturalearthdata.com).

## Geographic coverage

### Description

**Figure 1.**
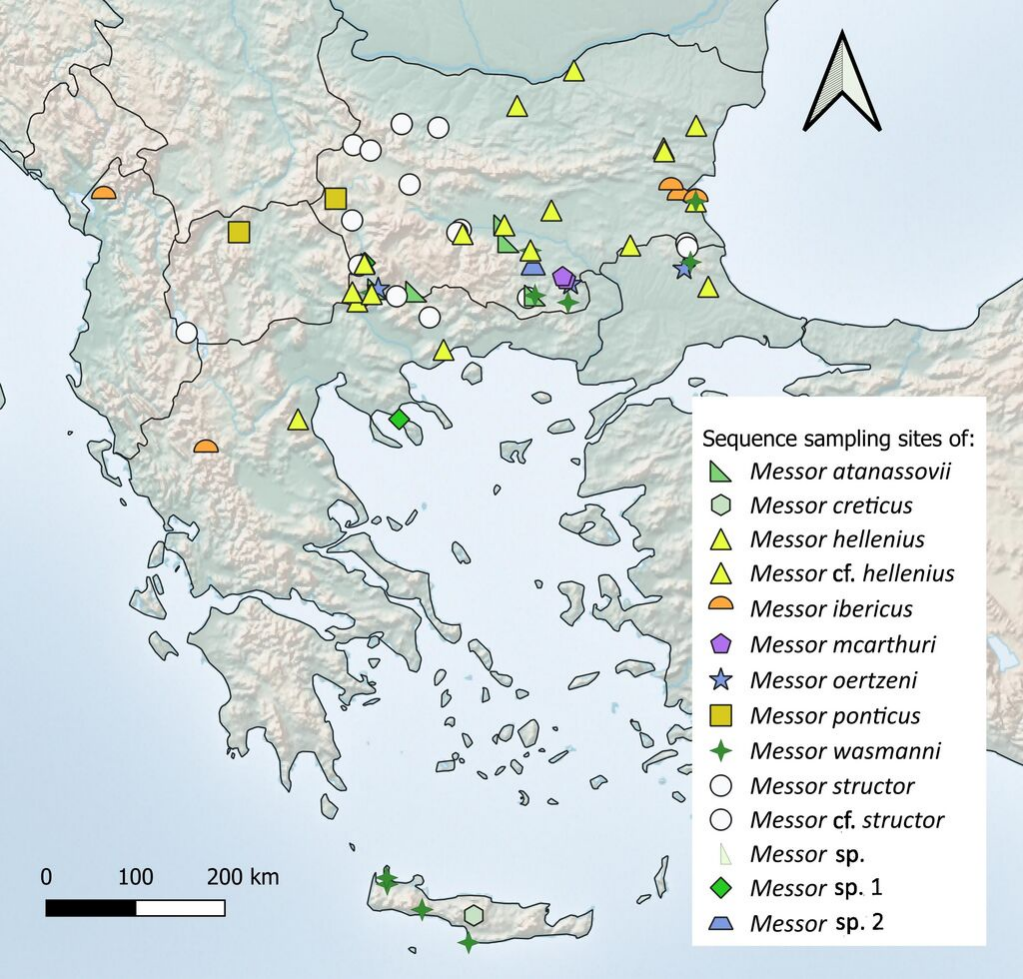
Map of sequence sampling sites.

The specimens used in this study were recently collected, primarily from Bulgaria (61), with additional samples from Greece (13), Türkiye (4), Albania (1) and North Macedonia (2) to ensure broader genetic and geographic representation (Fig. 1).

### Coordinates

 and Latitude: min. 34.931 max. 43.768 Latitude; and Longitude: min. 19.577 max. 27.794 Longitude.

## Taxonomic coverage

### Taxa included

**Table:** 

Rank	Scientific Name	
subfamily	Myrmicinae Lepeletier de Saint-Fargeau, 1835	
genus	*Aphaenogaster* Mayr, 1853	
species	*Aphaenogaster festae* Emery, 1915	
genus	*Messor* Forel, 1890	
species	*Messor atanassovii* Atanassov, 1982	
species	*Messor creticus* Salata & Borowiec, 2019	
species	*Messor hellenius* Agosti & Collingwood, 1987	
species	*Messor ibericus* Santschi, 1931	
species	*Messor mcarthuri* Steiner, Csősz, Markó, Gamisch, Rinnhofer, Folterbauer, Hammerle, Stauffer, Arthofer & Schlick-Steiner, 2018	
species	*Messor oertzeni* Forel, 1910	
species	*Messor ponticus* Steiner, Csősz, Markó, Gamisch, Rinnhofer, Folterbauer, Hammerle, Stauffer, Arthofer & Schlick-Steiner, 2018	
species	*Messor structor* (Latreille, 1798)	
species	*Messor wasmanni* Krausse, 1910	
species	Messor cf. structor	
species	*Messor* sp. 1	
species	*Messor* sp. 2	

## Usage licence

### Usage licence

Open Data Commons Attribution License

## Data resources

### Data package title

Collection of COI sequences from Bulgarian species of the genus *Messor*

### Resource link

https://doi.org/10.5883/DS-BGMESSOR

### Number of data sets

1

### Data set 1.

#### Data set name

Towards delimiting the diversity of *Messor* ants in Bulgaria using molecular data

#### Data format

dwc, xml, tsv, fasta

#### Description

The dataset constitutes a collection of sequences pertaining to Bulgarian species of the genus *Messor* (Hymenoptera, Formicidae). This dataset comprises all attributes and metadata in accordance with the BOLD rules and are available to the public via a Digital Object Identifier (DOI).

## Additional information

### Species delimitation and genetic diversity

**Figure 2.**
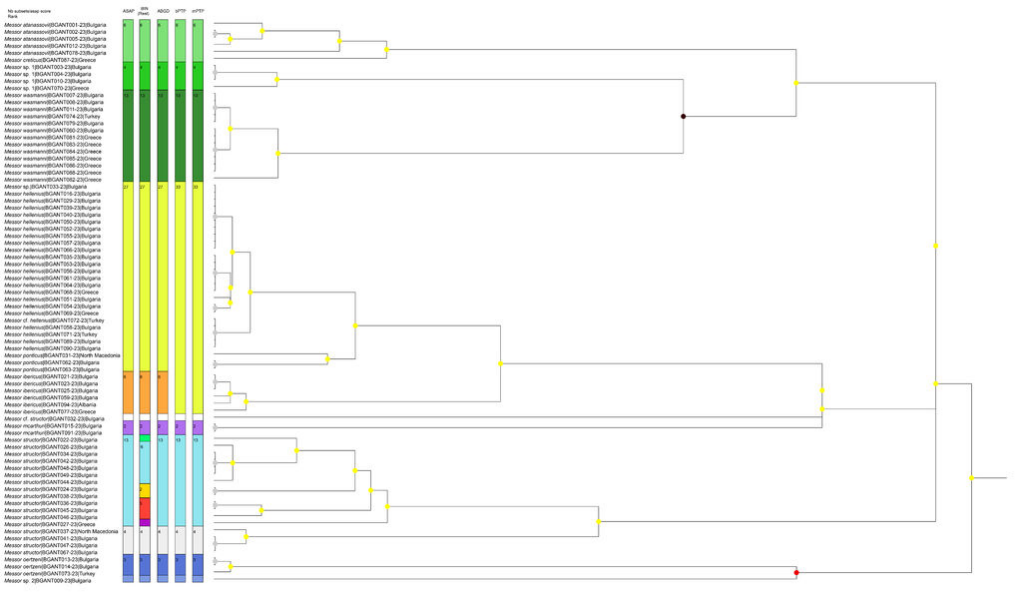
Results of species delimitation methods, based on DNA barcoding. Each vertical colour bar represents different delimitation schemes obtained with ASAP, RESL, ABGD, bPTP and mPTP methods, with the corresponding number of specimens. The tree is based on ASAP analysis, with nodes colour coded depending on their *p*-value (black: *p* < 0.001, red: *p* < 0.05, yellow: *p* > 0.1, grey: not applicable).

**Figure 3.**
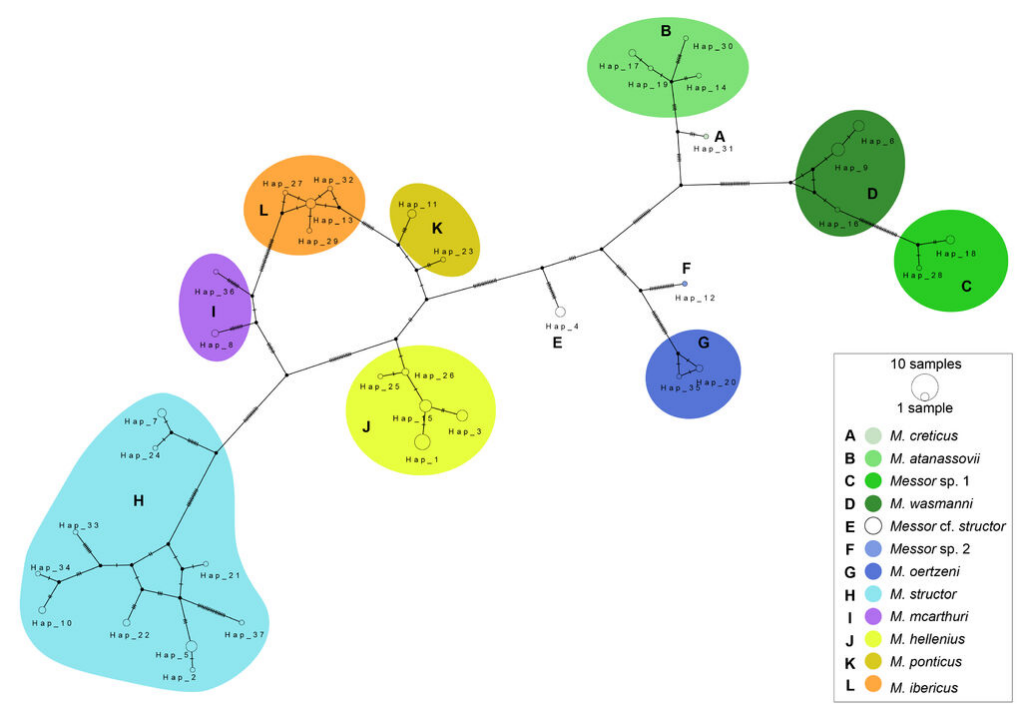
TCS haplotype network, based on COI sequences of *Messor* species. Each circle represents a unique haplotype; size corresponds to the number of individuals. Lines indicate single mutational steps. Species-specific colour and letter coding follow the phylogenetic tree.

A total of 84 COI sequences, representing 11 morphospecies and 10 to 15 molecular lineages (depending on the species delimitation method used), were analysed, including 80 newly-generated sequences. The lengths of the DNA barcodes ranged from 579 to 658 bp, with the majority (59 sequences) being 658 bp long (Fig. 2). Haplotype network analysis of all sequences revealed 35 distinct haplotypes, which clustered coherently (Fig. 3).

***Messor instabilis* species group**

The morphological similarity between *Messor atanassovii* and *M. creticus* is supported by low genetic distance observed in the delimitation analyses (K2P 1.92%) (Suppl. material 1). These findings may indicate a relatively recent divergence between the two species, followed by geographic isolation and ecological differentiation. However, despite their overall closeness, *M. atanassovii* and *M. creticus* consistently differ in stable morphological traits. Specifically, in *M. atanassovii*, the occipital area and vertex of the head bear 12–20 large setae, whereas in *M. creticus*, the number is always lower, never exceeding nine. In addition, unlike *M. creticus*, which is restricted to the mountain regions of Crete, *M. atanassovii* is a thermophilous lowland species found in southern Bulgaria, Central Macedonia and some of the Ionian Islands (Borowiec and Salata 2025). An ongoing research into the evolutionary history of this divergence will clarify the timing and mechanisms underlying this particular event.

Specimens from four nest samples — one from Central Macedonia in Greece and three from south-western Bulgaria — designated in this study as *Messor* sp. 1, exhibited morphological traits characteristic of both *Messor atanassovii* and *M. wasmanni*, specifically the setosity of the former and the larger size of the latter. However, all species delimitation analyses strongly supported their separation from both species and indicated a closer genetic affinity to *M. wasmanni*, with K2P distances of 6.28% and 5%, respectively (Suppl. material 1). Currently, *M. atanassovii* and *M. wasmanni* are the only known representatives of the *instabilis* group in this region. Whether the specimens designated as *Messor* sp. 1 represent cases of hybridogenesis or belong to a distinct species will be investigated in a future study.

The most widespread species of the *instabilis* group, *Messor wasmanni*, is represented in this study by a larger number of specimens (13) from the widest geographical range — spanning Bulgaria, Türkiye and Greece (including Crete). It exhibits an intraspecific genetic distance up to 0.81% (mean: 0.18%) (Suppl. material 1).

***Messor structor* species group**

Species delimitation analyses were consistent in supporting the distinctiveness of *Messor oertzeni*, *M. mcarthuri* and unidentified species close to *M. oertzeni* (named *Messor* sp. 2), as well as one molecular lineage within *Messor structor* represented by four sequences — three from western Bulgaria and one from North Macedonia. Further evaluation is also needed for a single sequence (BGANT032-23) obtained from a nest sample in the western Balkan Mountains (Vrachanski Balkan). Although this specimen is morphologically similar to *M. structor* and clearly separated from all recognised taxa in the analyses, the small sample size and absence of reproductive specimens make its taxonomic affinity still unclear.

*Messor ibericus* was consistently recognised in distance-based methods (ASAP, RESL, ABGD) and the coalescent-based method GMYC, exhibiting an intraspecific genetic distance up to 0.46% (mean 0.2%), but not in the tree-based methods bPTP and mPTP. This discrepancy can be attributed to differences in methodological assumptions and sensitivity to genetic variation (Hubert et al. 2024). It should be noted that our study analysed only the worker caste of *M. ibericus*, which, as recently shown by Juvé et al. (2025b), are hybrids with *M. structor*. Nevertheless, since they inherit the COI marker from the maternal lineage, the genetic patterns observed in our study remain consistent with their maternal identity.

Previous studies investigating *Messor structor* across its broad geographic distribution — spanning Austria, Bulgaria, Czechia, France, Hungary, Romania and Slovenia — revealed the existence of multiple mitochondrial lineages within the species (Schlick-Steiner et al. 2006, Steiner et al. 2018, Strohmaier et al. 2025). These earlier findings align closely with the results of the present study, which detected high intraspecific genetic diversity (with K2P distance from 0 to 5.07%, mean 2.58%) and identified ten haplotypes (Fig. 3, Suppl. material 1). While several species delimitation methods (ASAP, ABGD, bPTP, mPTP) recognised two molecular lineages, the RESL algorithm distinguished five BINs within *M. structor*, further supporting the presence of deep genetic structuring, with multiple lineages and haplotypes suggesting potential cryptic diversity across its range or long-term population isolation within the species.

Only the GMYC method succeeded in separating *M. hellenius* and *M. ponticus* (*Suppl. material 1*). The intraspecific genetic distance between 28 newly-generated sequences of both species from Bulgaria, North Macedonia and Greece range from 0 to 1.63% (Suppl. material 1). This result highlights the need to incorporate additional molecular data to clarify whether the morphological similarity observed by Borowiec and Salata (2025) truly reflects intraspecific variation or indicates the presence of subtle genetic structuring between closely-related species.

### Phylogenetic remarks

**Figure 4.**
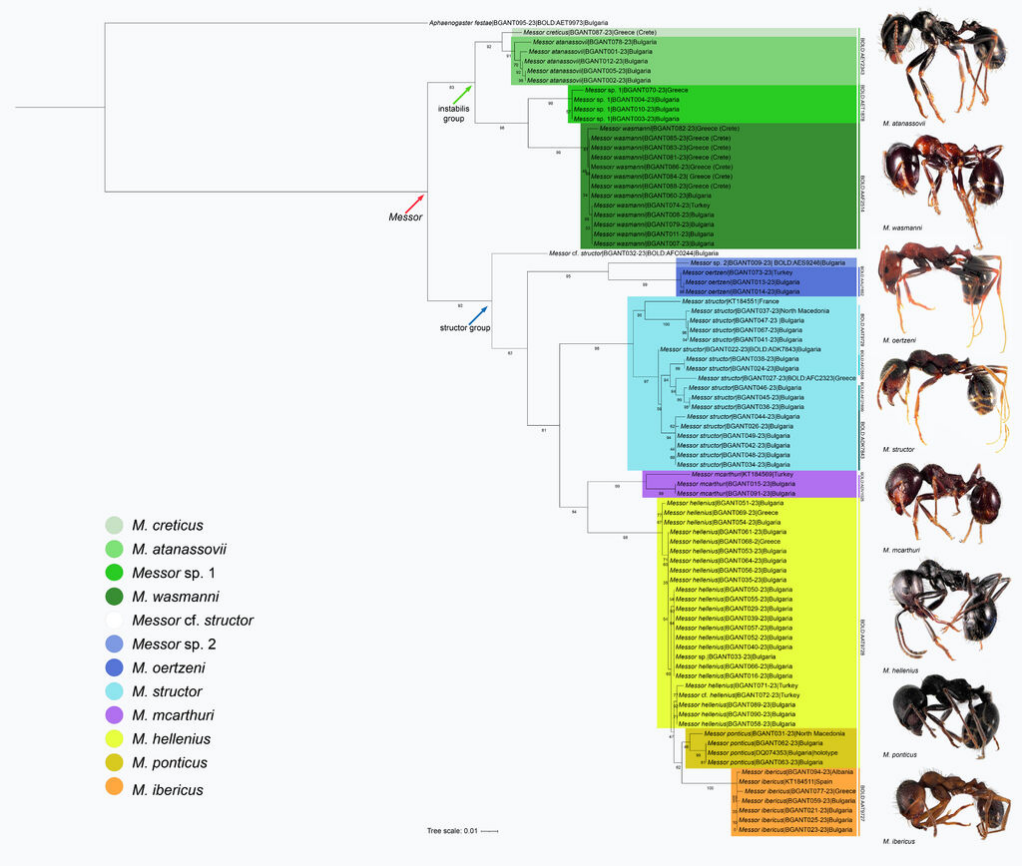
Phylogenetic tree based on the Maximum Likelihood analysis of COI gene fragments of representatives of genus *Messor*. Nodal support is assessed by bootstrap values. High nodal support for bootstrap values (BS) > 90%, moderately good support for BS > 70–90%.

The examined species fall into two well-defined species groups, *instabilis* and *structor* (*Fig. 4*). Both the *instabilis* (0.97 PP, 83% BS) and *structor* (0.90 PP, 92% BS) clades were consistently recovered across all phylogenetic analyses, receiving moderate to strong support (Fig. 4, Suppl. material 1).

Within the *instabilis* group, *Messor creticus* — included in the study to enhance both geographic and phylogenetic representation — was recovered as a sister taxon to *M. atanassovii* (1.00 PP, 92% BS), with very strong support, a finding that aligns well with morphological observations. Additionally, *Messor* sp. 1, which exhibits morphological traits intermediate between *M. atanassovii* and *M. wasmanni*, clusters closely with *M. wasmanni* with very strong support (1.00 PP, 96% BS).

In the *structor* group, *Messor hellenius* and *M. oertzeni* are nested within the clade, with *M. oertzeni* occupying a basal position (0.97 PP, 82% BS) supported by moderate to strong values. Their assignment to the *structor* group is in agreement with previous studies (Borowiec and Salata 2025, Juvé et al. 2025a). The specimen designated as "Messor cf. structor” (BGANT032-23) is resolved as a distinct lineage within the group, supported with strong confidence (0.99 PP, 92% BS).

The taxon labelled as "*Messor* sp. 2" is placed as sister to *M. oertzeni* (1.00 PP, 95% BS), supported with very strong confidence, consistent with morphological characteristics observed in the nest sample.

*Messor structor* itself exhibits considerable genetic diversity, reflected in the phylogenetic analyses by multiple well-supported lineages within its clade. Such diversity is expected given the species’ broad distribution across Europe and likely corresponds to population-level differentiation.

The recently described *M. mcarthuri* (0.93 PP, 84% BS) is recovered as sister to the clade comprising *M. hellenius*, *M. ponticus* and *M. ibericus*, consistent with the topology of the latter two species reported in Juvé et al. (2025a). In the Bayesian Inference (BI) phylogenetic tree, *M. ibericus* appears as sister to the *M. hellenius* + *M. ponticus* clade. In contrast, in the ML tree, *M. ibericus* clusters with *M. ponticus*, while *M. hellenius* appears polyphyletic, albeit with weak bootstrap support, which could be due to the limitations of the single-locus dataset (Fig. 4, Suppl. material 1). Nonetheless, the Balkan *M. hellenius* exhibits relatively high genetic diversity, a pattern that warrants further investigation.

### Conclusion

Our results provide valuable reference material for future research and highlight the importance of applying integrative taxonomic approaches to studies of ant biodiversity. Furthermore, DNA barcoding can contribute to elucidating the phylogenetic relationships within the genus, offering insights into evolutionary lineages.

However, caution should be exercised when inferring species identification solely on morphology or solely on the COI sequence data for taxonomically challenging species, without taking into account biological and ecological data (e.g. interactions between neighbouring colonies of different morphospecies, differences in nest structure, foraging systems or diurnal activity). Deeper genetic studies are required to explain the observed tendency of some species to form hybridogenetic populations with distinct morphology, which may lead to the description of new species.

## Supplementary Material

30056884-0B5D-529E-BDEA-ABAFF8AA1FB4 10.3897/BDJ.13.e168586.suppl1 Supplementary material 1 Distance analyses, GMYC summary and Bayesian Inference tree Data type genomic, phylogenetic File: oo_1444534.pdf https://binary.pensoft.net/file/1444534 Albena Lapeva-Gjonova, Monika Pramatarova, Lech Borowiec, Ilia Gjonov, Rumyana Kostova, Rostislav Bekchiev, Simeon Borissov
