# Supplementary material for: DNA barcoding of Messor ants of Bulgaria with insights into their taxonomic diversity
Source: Biodivers Data J. 2025 Nov 4;13:e168586. doi: 10.3897/BDJ.13.e168586 (PMC12606073; doi:10.3897/BDJ.13.e168586)
Supplement: Supplementary material 1 — Distance analyses, GMYC summary and Bayesian Inference tree [file bdj-13-e168586-s001.pdf]

Supplementary materials. COI genetic distance (%) within studied *Messor* species

species	n	min	max
Messor atanassovii	5	0	1.41
Messor sp. 1 (near *M. wasmanni*)	4	0	0.61
Messor wasmanni	13	0	0.81
Messor oertzeni	3	0	0.15
Messor structor	17	0	5.07
Messor mcarthuri	2	0	0.00
Messor hellenius	24	0	0.99
Messor ponticus	4	0	1.38
Messor ibericus	7	0	0.46

COI genetic distance (%) between *Messor* species under the Kimura 2-parameter model. Values in brackets represent the mean within species distance. NA/n.a.: not available.

N	species	1	2	3	4	5	6	7	8	9	10	11	12
1.	*M. creticus*	NA (n.a)											
2.	*M. atanassovii*	1.92	NA (0.74)										
3.	*Messor* sp. 1 (near *M. wasmanni*)	5.98	6.28	NA (0.31)									
4.	*M. wasmanni*	5.80	9.58	5.00	NA (0.18)								
5.	*M. oertzeni*	9.05	9.40	10.71	10.24	NA (0.10)							
6.	*M. structor*	8.99	9.18	10.20	10.18	9.22	NA (2.57)						
7.	*M. mcarthuri*	9.73	9.98	10.14	11.55	8.89	8.49	NA (0)					
8.	*M. hellenius*	9.50	9.66	10.12	10.88	9.13	8.48	6.47	NA (0.35)				
9.	*M. ponticus*	9.56	9.64	10.67	11.28	9.40	8.57	7.01	1.63	NA (0.83)			
10.	*M. ibericus*	9.26	9.48	10.29	10.72	8.93	8.40	6.73	3.20	3.30	NA (0.20)		
11.	*Messor* cf. *structor*	7.00	7.61	8.83	9.50	7.94	7.79	7.37	6.19	6.39	6.50	NA (n.a)	
12.	*Messor* sp. 2 (near *M. oertzeni*)	10.61	10.64	11.62	11.73	5.72	9.81	9.11	8.88	8.84	9.21	7.38	NA (n.a)

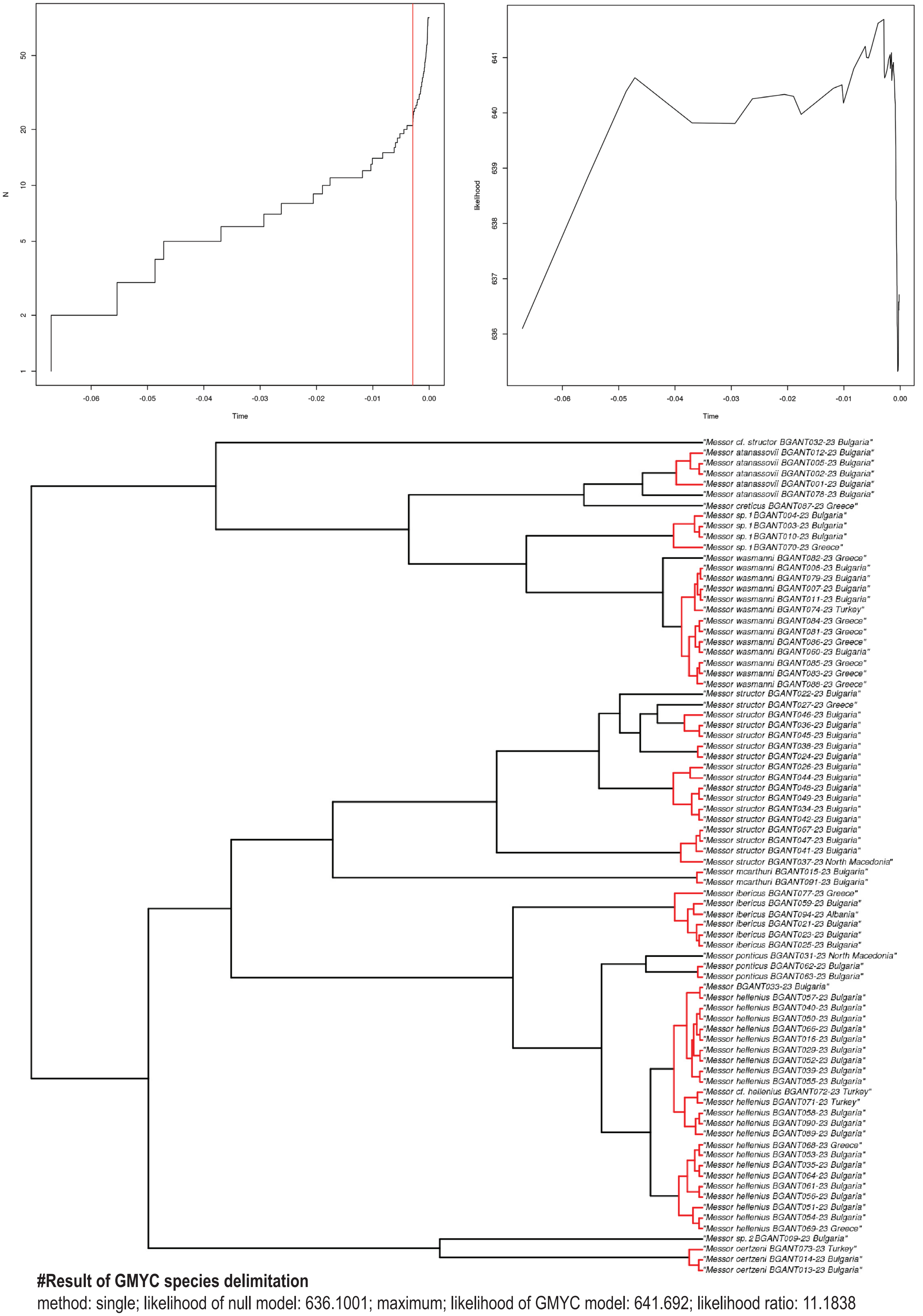

#Result of GMYC species delimitation

method: single; likelihood of null model: 636.1001; maximum; likelihood of GMYC model: 641.692; likelihood ratio: 11.1838 result of LR test: 0.003727933**; number of ML clusters: 13; confidence interval: 3-23; number of ML entities: 21; confidence interval: 3-42; threshold time: -0.002905204

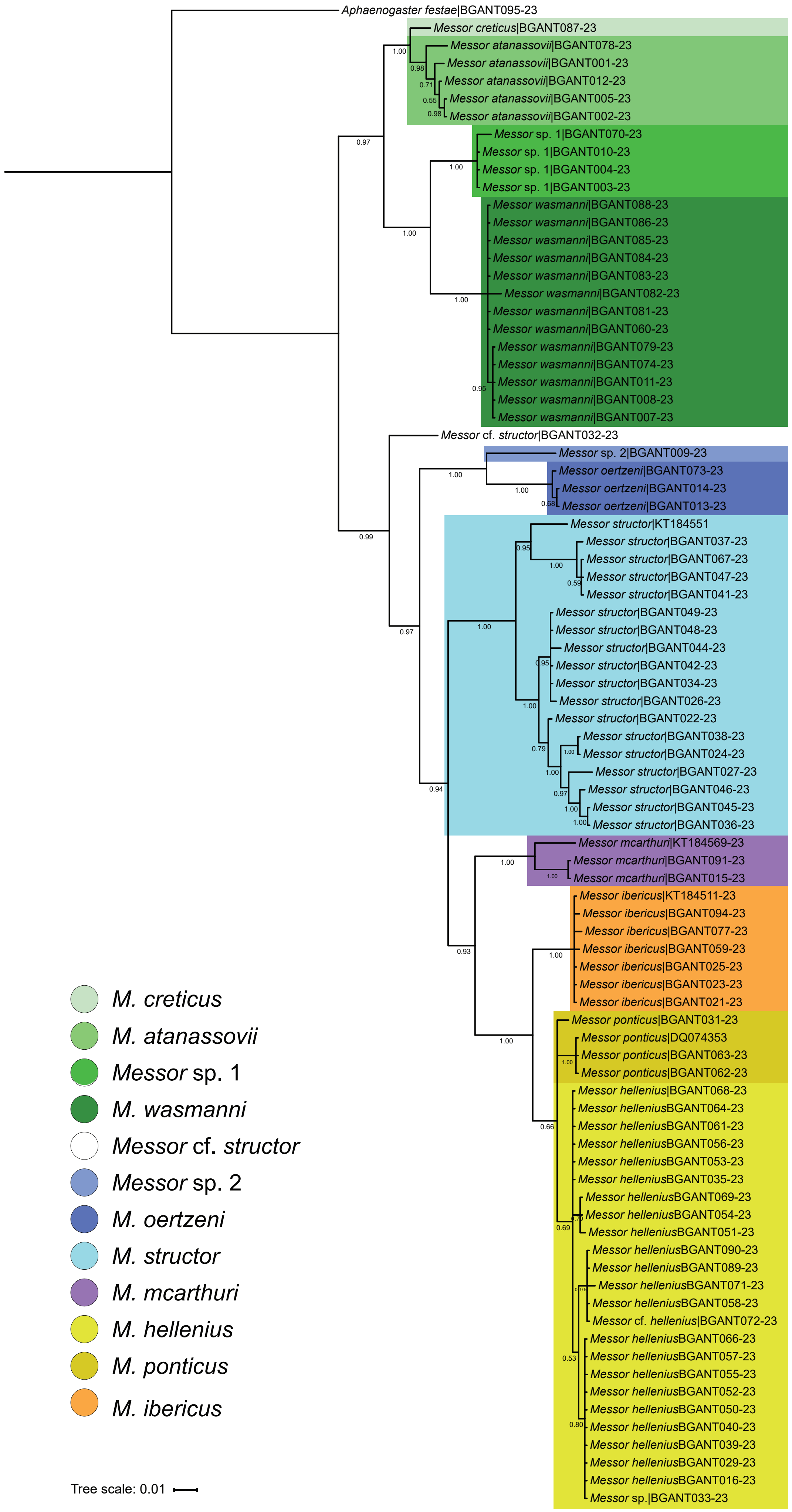

Phylogenetic tree based on Bayesian inference (BI) analysis of COI gene fragment. Nodal support is given as posterior probability values. We considered significantly high nodal support for posterior probabilities (PP) > 0.95, and moderately good support for PP > 0.90–0.94.
